# The cocrystal μ-oxalato-κ^4^ *O* ^1^,*O* ^2^:*O* ^1′^,*O* ^2′^-bis­(aqua­(nitrato-κ*O*){[1-(2-pyridyl-κ*N*)eth­ylidene]hydrazine-κ*N*}copper(II)) μ-oxalato-κ^4^ *O* ^1^,*O* ^2^:*O* ^1′^,*O* ^2′^-bis­((methanol-κ*O*)(nitrato-κ*O*){[1-(2-pyridyl-κ*N*)eth­ylidene]hydrazine-κ*N*}copper(II)) (1/1)

**DOI:** 10.1107/S1600536808024550

**Published:** 2008-08-06

**Authors:** Madina Diallo, Farba Bouyagui Tamboura, Mohamed Gaye, Aliou Hamady Barry, Youssouph Bah

**Affiliations:** a Département de Chimie, Faculté des Sciences et Techniques, Université Cheikh Anta Diop, Dakar, Senegal; b Département de Chimie, Faculté des Sciences, Université de Nouakchott, Nouakchott, Mauritania; c Département de Chimie, Faculté des Sciences, Université de Conakry, Conakry, Guinea

## Abstract

The title cocrystal, [Cu_2_(C_2_O_4_)(NO_3_)_2_(C_7_H_9_N_3_)_2_(H_2_O)_2_][Cu_2_(C_2_O_4_)(NO_3_)_2_(C_7_H_9_N_3_)_2_(CH_4_O)_2_], is a 1:1 cocrystal of two centrosymmetric Cu^II^ complexes with oxalate dianions and Schiff base ligands. In each mol­ecule, the Cu^II^ centre is in a distorted octa­hedral *cis*-CuN_2_O_4_ environment, the donor atoms of the *N*,*N*′-bidentate Schiff base ligand and the bridging *O*,*O*′-bidentate oxalate group lying in the equatorial plane. In one mol­ecule, a monodentate nitrate anion and a water mol­ecule occupy the axial sites, and in the other, a monodentate nitrate anion and a methanol mol­ecule occupy these sites. In the crystal structure, inter­molecular N—H⋯O, O—H⋯O and N—H⋯N hydrogen bonds link the mol­ecules into a network. Weak intra­molecular N—H⋯O inter­actions are also observed.

## Related literature

For related structures: see Kelly *et al.* (2005 ▶); Bulut *et al.* (2005 ▶); Moreno *et al.* (2007 ▶); Du *et al.* (2007 ▶).
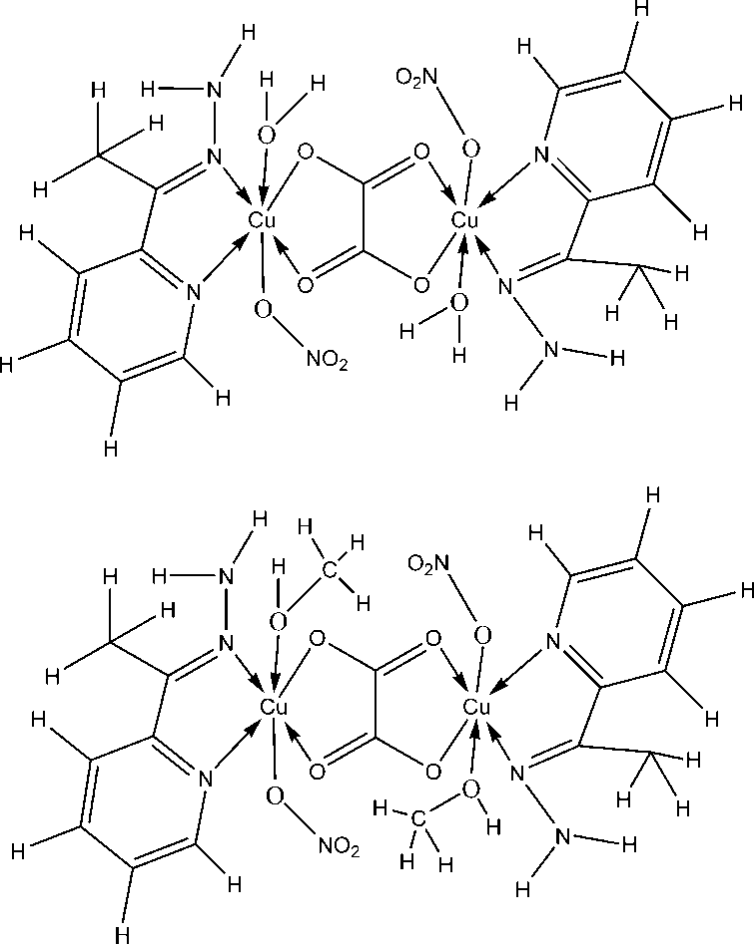


## Experimental

### 

#### Crystal data

[Cu_2_(C_2_O_4_)(NO_3_)_2_(C_7_H_9_N_3_)_2_(H_2_O)_2_][Cu_2_(C_2_O_4_)(NO_3_)_2_(C_7_H_9_N_3_)_2_(CH_4_O)_2_]
*M* *_r_* = 1319.04
Triclinic, 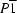

*a* = 9.8358 (9) Å
*b* = 12.3773 (10) Å
*c* = 12.7136 (10) Å
α = 103.704 (4)°
β = 112.573 (4)°
γ = 107.821 (4)°
*V* = 1245.15 (18) Å^3^
*Z* = 1
Mo *K*α radiation
μ = 1.79 mm^−1^
*T* = 173 (2) K
0.03 × 0.02 × 0.02 mm

#### Data collection

Nonius KappaCCD diffractometer
Absorption correction: none
11826 measured reflections
4872 independent reflections
3644 reflections with *I* > 2σ(*I*)
*R*_int_ = 0.057

#### Refinement

*R*[*F* ^2^ > 2σ(*F* ^2^)] = 0.059
*wR*(*F* ^2^) = 0.112
*S* = 1.06
4872 reflections
380 parameters
6 restraints
H atoms treated by a mixture of independent and constrained refinement
Δρ_max_ = 0.92 e Å^−3^
Δρ_min_ = −0.54 e Å^−3^

### 

Data collection: *COLLECT* (Nonius, 1998 ▶); cell refinement: *DENZO* (Nonius, 1998 ▶); data reduction: *DENZO*; program(s) used to solve structure: *SHELXS97* (Sheldrick, 2008 ▶); program(s) used to refine structure: *SHELXL97* (Sheldrick, 2008 ▶); molecular graphics: *PLATON* (Spek, 2003 ▶); software used to prepare material for publication: *SHELXL97*.

## Supplementary Material

Crystal structure: contains datablocks I, global. DOI: 10.1107/S1600536808024550/hb2759sup1.cif

Structure factors: contains datablocks I. DOI: 10.1107/S1600536808024550/hb2759Isup2.hkl

Additional supplementary materials:  crystallographic information; 3D view; checkCIF report

## Figures and Tables

**Table 1:** Selected bond lengths (Å)

Cu1—N1	1.966 (4)
Cu1—O1	1.986 (3)
Cu1—N2	1.991 (4)
Cu1—O2	2.000 (3)
Cu1—O3	2.252 (4)
Cu1—O4	2.610 (4)
Cu2—N5	1.955 (4)
Cu2—O7	1.966 (3)
Cu2—O8	1.981 (3)
Cu2—N6	1.994 (4)
Cu2—O9	2.337 (3)
Cu2—O10	2.541 (4)

**Table 2:** Hydrogen-bond geometry (Å, °)

*D*—H⋯*A*	*D*—H	H⋯*A*	*D*⋯*A*	*D*—H⋯*A*
O3—H18⋯O11^i^	0.96 (2)	2.11 (3)	3.048 (7)	164 (5)
O3—H18⋯O12^i^	0.96 (2)	2.30 (4)	3.084 (6)	138 (5)
O3—H18⋯N8^i^	0.96 (2)	2.53 (2)	3.471 (6)	167 (5)
O3—H19⋯O9^ii^	0.95 (2)	1.85 (2)	2.777 (5)	165 (5)
N3—H20⋯O1	0.95 (2)	2.53 (7)	3.014 (6)	112 (5)
N3—H21⋯O12	0.97 (2)	2.44 (7)	3.150 (7)	130 (7)
N7—H22⋯O6	0.96 (2)	2.35 (4)	3.131 (6)	138 (5)
N7—H23⋯N3	0.95 (2)	2.43 (5)	3.196 (7)	138 (5)
N7—H23⋯O7	0.95 (2)	2.59 (6)	3.091 (5)	113 (5)
O9—H24⋯O6^iii^	0.84	1.89	2.731 (5)	174
O9—H24⋯N4^iii^	0.84	2.57	3.367 (6)	158
O9—H24⋯O5^iii^	0.84	2.56	3.171 (6)	130

Symmetry codes: (i) 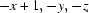; (ii) 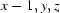; (iii) 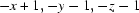.

**Fig. 1.**
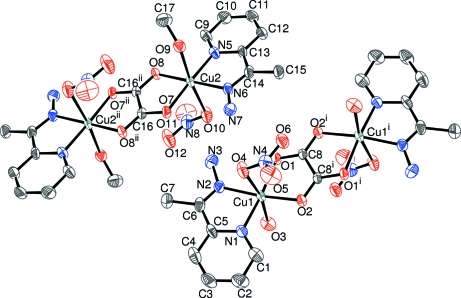
The molecular structure of (I) (H atoms are omitted for clarity). Displacement ellipsoids are plotted at the 50% probability level. Symmetry codes: (i) -x, -y-1, -z-1; (ii) 2-x, -y, -z.

**Fig. 2.**
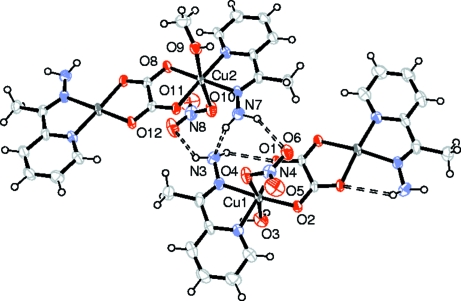
Part of the packing in (I) showing hydrogen bonds as broken lines.

## supplementary crystallographic information

### Comment

The molecular structure of the title compound, (I), (Fig. 1) contains two centrosymmetric binuclear Cu^II^ complexes (A and B) with the same Schiff base ligand. The coordination spheres around Cu^II^ in both A and B are slightly distorted octahedra (Table 1), with the coordination plane of each Cu^II^ formed by the N_2_O_2_donor atoms of the Schiff base N_2_ and the oxalate O_2_. The axial positions in A are occupied by an O—NO_2_ ion and a water molecule whereas in B these positions are occupied by a O-NO_2_ ion and a CH_3_OH molecule. The in-plane Cu—O distances are in the range 1.966 (3)–2.000 (3) Å with Cu—N distances 1.955 (4)–1.994 (4) Å, which are slightly larger than distances observed in other Cu^II^ coordination complexes of the same Schiff base ligand (Kelly et al <i\>., 2005).  The elongation of the Cu—O—NO_2_ and Cu—O(water) or Cu—O(methanol) axial bonds [2.610 (4) and 2.251 (4) Å in A and 2.541 (4) and 2.338 (3) Å in  B] clearly indicate the usual Jahn Teller distortion of the Cu^II^ as has been found previously (Bulut et al <i\>., 2005; Moreno et al <i\>., 2007; Du et al <i\>., 2007).  The basal bond angles O–Cu–O and N–Cu–N are less then 90° [N1–Cu1–N2 = 80.63 (18) ° and N5–Cu2–N6 = 81.50 (17) °; O1–Cu1–O2 = 84.01 (13) ° and O7–Cu2–O8 = 85.09 (17) °] whereas the O–Cu–N angles are largely superior to 90° [N1–Cu1–O2 = 98.76 (16) ° and N2–Cu1–O1 =95.46 (16) ° in A; N5–Cu2–O8 = 95.29 (15) ° and N6–Cu2–O7 =97.93 (15) ° in B]. The axial bonds angles O(water)–Cu1–O—NO_2_ and O(methanol)–Cu2–O—NO_2_ are also less than the ideal value of 180° [168.34 (14) ° in A and 173.96 (12) ° in B].  A network of hydrogren bonds (Table 2) completes the structure.

### Experimental

To a mixture of 0.324 g (1.0 mmol) of the ligand and 50 ml of methanol was added dropwise a solution of 0.463 g (2.0 mmol) of copper nitrate dihydrate in 10 ml of methanol. The resulting mixture was stirred under reflux for 120 min. After cooling the solution was left for slow evaporation and the title compound was obtained in good yield (0.620 g; 94.00°). IR (cm^-1^,KBr): 1655, 1625, 1603, 1585, 1484, 1375, 1327, 1030, 829, 783, 699. Analysis calculated for C_34_H_48_N_16_O_24_Cu_4_: C 30.96, H 3.67, N 16.99 °; found: C 30.94, H 3.69, N 16.95 °.  Gren prisms of (I) were obtained from slow evaporation of a dimethylformamide solution.

### Refinement

All water H atoms and amine H atoms of the bidentate Schiff base ligand were located from the difference fourier map and refined. The water O—H and amine N—H distances were restrained to be 0.96 Å, with s.u's of 0.02 Å. Others H atoms were placed geometrically and refined with a riding model. *U*_iso_(H) for H was assigned as 1.2 *U*_eq_ of the attached C or N atoms (1.5 for methyl C atoms).

### Crystal data

**Table:**

[Cu_2_(C_2_O_4_)(NO_3_)_2_(C_7_H_9_N_3_)_2_(H_2_O)_2_] [Cu_2_(C_2_O_4_)(NO_3_)_2_(C_7_H_9_N_3_)_2_(CH_4_O)_2_]	*Z* = 1
*M**_r_* = 1319.04	*F*_000_ = 672
Triclinic, *P*1	*D*_x_ = 1.759 Mg m^−^^3^
Hall symbol: -P 1	Mo *K*α radiation λ = 0.71073 Å
*a* = 9.8358 (9) Å	Cell parameters from 3872 reflections
*b* = 12.3773 (10) Å	θ = 1.0–26.0º
*c* = 12.7136 (10) Å	µ = 1.79 mm^−^^1^
α = 103.704 (4)º	*T* = 173 (2) K
β = 112.573 (4)º	Prism, green
γ = 107.821 (4)º	0.03 × 0.02 × 0.02 mm
*V* = 1245.15 (18) Å^3^	

### Data collection

**Table:**

Nonius KappaCCD diffractometer	3644 reflections with *I* > 2σ(*I*)
Radiation source: fine-focus sealed tube	*R*_int_ = 0.057
Monochromator: graphite	θ_max_ = 26.0º
*T* = 173(2) K	θ_min_ = 1.9º
π [IS PI CORRECT?] scans	*h* = −12→11
Absorption correction: none	*k* = −14→15
11826 measured reflections	*l* = −15→15
4872 independent reflections	

### Refinement

**Table:**

Refinement on *F*^2^	Secondary atom site location: difference Fourier map
Least-squares matrix: full	Hydrogen site location: difmap and geom
*R*[*F*^2^ > 2σ(*F*^2^)] = 0.059	H atoms treated by a mixture of independent and constrained refinement
*wR*(*F*^2^) = 0.112	*w* = 1/[σ^2^(*F*_o_^2^) + (0.0157*P*)^2^ + 3.8674*P*] where *P* = (*F*_o_^2^ + 2*F*_c_^2^)/3
*S* = 1.07	(Δ/σ)_max_ = 0.009
4872 reflections	Δρ_max_ = 0.92 e Å^−^^3^
380 parameters	Δρ_min_ = −0.54 e Å^−^^3^
6 restraints	Extinction correction: none
Primary atom site location: structure-invariant direct methods	

### Special details

**Table:**

Geometry. All e.s.d.'s (except the e.s.d. in the dihedral angle between two l.s. planes) are estimated using the full covariance matrix. The cell e.s.d.'s are taken into account individually in the estimation of e.s.d.'s in distances, angles and torsion angles; correlations between e.s.d.'s in cell parameters are only used when they are defined by crystal symmetry. An approximate (isotropic) treatment of cell e.s.d.'s is used for estimating e.s.d.'s involving l.s. planes.
Refinement. Refinement of *F*^2^ against ALL reflections. The weighted *R*-factor *wR* and goodness of fit *S* are based on *F*^2^, conventional *R*-factors *R* are based on *F*, with *F* set to zero for negative *F*^2^. The threshold expression of *F*^2^ > σ(*F*^2^) is used only for calculating *R*-factors(gt) *etc*. and is not relevant to the choice of reflections for refinement. *R*-factors based on *F*^2^ are statistically about twice as large as those based on *F*, and *R*- factors based on ALL data will be even larger.

### Fractional atomic coordinates and isotropic or equivalent isotropic displacement parameters (Å^2^)

**Table:**

	*x*	*y*	*z*	*U*_iso_*/*U*_eq_	
Cu1	0.16109 (7)	−0.43205 (5)	−0.26049 (5)	0.02519 (16)	
Cu2	0.80107 (7)	−0.09303 (5)	−0.23584 (5)	0.02536 (16)	
O1	0.1705 (4)	−0.3628 (3)	−0.3847 (3)	0.0256 (7)	
O2	−0.0358 (4)	−0.5775 (3)	−0.4088 (3)	0.0256 (7)	
O3	0.0280 (5)	−0.3375 (4)	−0.1957 (4)	0.0411 (9)	
O4	0.3402 (5)	−0.5415 (4)	−0.2903 (3)	0.0421 (9)	
O5	0.2179 (6)	−0.7368 (4)	−0.4051 (4)	0.0650 (13)	
O6	0.2292 (5)	−0.6055 (3)	−0.4905 (3)	0.0406 (9)	
O7	0.8412 (4)	−0.1491 (3)	−0.0993 (3)	0.0260 (7)	
O8	0.9916 (4)	0.0660 (3)	−0.1073 (3)	0.0264 (7)	
O9	0.9512 (4)	−0.1775 (3)	−0.3019 (3)	0.0342 (8)	
H24	0.8965	−0.2413	−0.3686	0.08 (3)*	
O10	0.6195 (5)	−0.0053 (4)	−0.1877 (3)	0.0470 (10)	
O11	0.7199 (6)	0.1888 (4)	−0.0798 (5)	0.0727 (15)	
O12	0.7466 (6)	0.0644 (4)	0.0110 (4)	0.0639 (13)	
N1	0.1741 (5)	−0.4997 (4)	−0.1337 (4)	0.0303 (10)	
N2	0.3849 (5)	−0.3083 (4)	−0.1229 (4)	0.0306 (10)	
N3	0.4829 (6)	−0.2153 (5)	−0.1376 (5)	0.0483 (13)	
N4	0.2618 (5)	−0.6290 (4)	−0.3950 (4)	0.0343 (10)	
N5	0.7526 (5)	−0.0355 (4)	−0.3705 (4)	0.0287 (9)	
N6	0.5987 (5)	−0.2449 (4)	−0.3691 (3)	0.0277 (9)	
N7	0.5443 (6)	−0.3515 (4)	−0.3500 (4)	0.0345 (10)	
N8	0.6948 (6)	0.0835 (5)	−0.0856 (4)	0.0415 (12)	
C1	0.0535 (8)	−0.5935 (5)	−0.1446 (5)	0.0396 (13)	
H1	−0.0475	−0.6368	−0.2208	0.048*	
C2	0.0702 (9)	−0.6314 (5)	−0.0462 (6)	0.0503 (16)	
H2	−0.0178	−0.6988	−0.0545	0.060*	
C3	0.2192 (8)	−0.5670 (5)	0.0630 (5)	0.0472 (15)	
H3	0.2353	−0.5910	0.1308	0.057*	
C4	0.3432 (8)	−0.4695 (6)	0.0739 (5)	0.0468 (15)	
H4	0.4456	−0.4256	0.1490	0.056*	
C5	0.3192 (7)	−0.4341 (5)	−0.0259 (4)	0.0316 (12)	
C6	0.4383 (6)	−0.3281 (5)	−0.0245 (5)	0.0362 (13)	
C7	0.6073 (7)	−0.2464 (6)	0.0880 (5)	0.0503 (16)	
H7A	0.6882	−0.2226	0.0606	0.076*	
H7B	0.6354	−0.2921	0.1398	0.076*	
H7C	0.6076	−0.1718	0.1366	0.076*	
C8	0.0597 (6)	−0.4386 (4)	−0.4928 (4)	0.0221 (10)	
C9	0.8353 (7)	0.0747 (5)	−0.3624 (5)	0.0346 (12)	
H9	0.9293	0.1336	−0.2851	0.042*	
C10	0.7900 (7)	0.1081 (5)	−0.4626 (5)	0.0425 (14)	
H10	0.8512	0.1884	−0.4548	0.051*	
C11	0.6536 (7)	0.0212 (6)	−0.5740 (5)	0.0414 (14)	
H11	0.6201	0.0418	−0.6442	0.050*	
C12	0.5649 (7)	−0.0957 (5)	−0.5847 (5)	0.0374 (13)	
H12	0.4699	−0.1549	−0.6613	0.045*	
C13	0.6171 (6)	−0.1254 (5)	−0.4813 (4)	0.0292 (11)	
C14	0.5375 (6)	−0.2435 (5)	−0.4771 (4)	0.0288 (11)	
C15	0.3982 (6)	−0.3556 (5)	−0.5918 (5)	0.0392 (13)	
H15A	0.4234	−0.4267	−0.5971	0.059*	
H15B	0.3837	−0.3385	−0.6659	0.059*	
H15C	0.2969	−0.3744	−0.5871	0.059*	
C16	0.9575 (5)	−0.0615 (4)	0.0021 (4)	0.0217 (10)	
C17	1.0924 (8)	−0.0986 (6)	−0.2984 (6)	0.0484 (15)	
H17A	1.0615	−0.0565	−0.3535	0.073*	
H17B	1.1430	−0.1476	−0.3263	0.073*	
H17C	1.1708	−0.0368	−0.2131	0.073*	
H18	0.107 (6)	−0.277 (4)	−0.112 (3)	0.058 (19)*	
H19	−0.001 (7)	−0.277 (4)	−0.220 (5)	0.050 (17)*	
H20	0.418 (8)	−0.186 (6)	−0.190 (5)	0.09 (3)*	
H21	0.570 (7)	−0.154 (6)	−0.055 (4)	0.12 (3)*	
H22	0.437 (4)	−0.406 (4)	−0.420 (4)	0.055 (18)*	
H23	0.559 (8)	−0.332 (6)	−0.268 (3)	0.07 (2)*	

### Atomic displacement parameters (Å^2^)

**Table:**

	*U*^11^	*U*^22^	*U*^33^	*U*^12^	*U*^13^	*U*^23^
Cu1	0.0265 (3)	0.0263 (3)	0.0162 (3)	0.0107 (3)	0.0062 (3)	0.0068 (3)
Cu2	0.0265 (3)	0.0248 (3)	0.0155 (3)	0.0102 (3)	0.0045 (3)	0.0052 (2)
O1	0.0230 (18)	0.0232 (17)	0.0179 (17)	0.0062 (15)	0.0039 (15)	0.0045 (14)
O2	0.0258 (18)	0.0257 (18)	0.0199 (17)	0.0103 (15)	0.0074 (15)	0.0088 (14)
O3	0.056 (3)	0.041 (2)	0.042 (2)	0.033 (2)	0.028 (2)	0.020 (2)
O4	0.042 (2)	0.048 (2)	0.024 (2)	0.024 (2)	0.0072 (18)	0.0050 (18)
O5	0.096 (4)	0.041 (3)	0.067 (3)	0.034 (3)	0.045 (3)	0.023 (2)
O6	0.042 (2)	0.043 (2)	0.027 (2)	0.0173 (19)	0.0132 (18)	0.0070 (17)
O7	0.0223 (18)	0.0228 (17)	0.0191 (17)	0.0053 (15)	0.0038 (15)	0.0042 (14)
O8	0.0283 (19)	0.0267 (18)	0.0164 (16)	0.0106 (16)	0.0054 (15)	0.0082 (14)
O9	0.035 (2)	0.038 (2)	0.026 (2)	0.0173 (18)	0.0145 (17)	0.0090 (18)
O10	0.041 (2)	0.052 (3)	0.029 (2)	0.021 (2)	0.0076 (19)	0.0044 (19)
O11	0.095 (4)	0.042 (3)	0.098 (4)	0.040 (3)	0.057 (4)	0.025 (3)
O12	0.071 (3)	0.078 (3)	0.027 (2)	0.039 (3)	0.012 (2)	0.010 (2)
N1	0.031 (2)	0.035 (2)	0.025 (2)	0.017 (2)	0.013 (2)	0.0117 (19)
N2	0.027 (2)	0.034 (2)	0.023 (2)	0.012 (2)	0.011 (2)	0.0039 (19)
N3	0.034 (3)	0.044 (3)	0.042 (3)	−0.001 (3)	0.015 (3)	0.010 (3)
N4	0.034 (3)	0.034 (3)	0.040 (3)	0.022 (2)	0.018 (2)	0.013 (2)
N5	0.034 (2)	0.032 (2)	0.022 (2)	0.019 (2)	0.012 (2)	0.0122 (19)
N6	0.025 (2)	0.031 (2)	0.020 (2)	0.013 (2)	0.0076 (19)	0.0025 (18)
N7	0.033 (3)	0.027 (2)	0.032 (3)	0.006 (2)	0.014 (2)	0.008 (2)
N8	0.038 (3)	0.045 (3)	0.040 (3)	0.026 (2)	0.017 (2)	0.007 (2)
C1	0.052 (4)	0.041 (3)	0.035 (3)	0.028 (3)	0.023 (3)	0.017 (3)
C2	0.069 (5)	0.035 (3)	0.052 (4)	0.019 (3)	0.033 (4)	0.026 (3)
C3	0.067 (4)	0.040 (3)	0.036 (3)	0.023 (3)	0.022 (3)	0.025 (3)
C4	0.051 (4)	0.057 (4)	0.029 (3)	0.032 (3)	0.011 (3)	0.016 (3)
C5	0.038 (3)	0.043 (3)	0.018 (2)	0.029 (3)	0.011 (2)	0.009 (2)
C6	0.028 (3)	0.043 (3)	0.023 (3)	0.017 (3)	0.008 (2)	−0.002 (2)
C7	0.034 (3)	0.066 (4)	0.035 (3)	0.015 (3)	0.011 (3)	0.014 (3)
C8	0.019 (2)	0.024 (2)	0.017 (2)	0.010 (2)	0.006 (2)	0.005 (2)
C9	0.042 (3)	0.043 (3)	0.025 (3)	0.025 (3)	0.015 (3)	0.018 (2)
C10	0.047 (4)	0.043 (3)	0.046 (3)	0.021 (3)	0.025 (3)	0.028 (3)
C11	0.043 (3)	0.064 (4)	0.034 (3)	0.031 (3)	0.021 (3)	0.032 (3)
C12	0.034 (3)	0.053 (4)	0.026 (3)	0.022 (3)	0.013 (3)	0.018 (3)
C13	0.024 (3)	0.044 (3)	0.019 (2)	0.021 (2)	0.007 (2)	0.010 (2)
C14	0.023 (3)	0.032 (3)	0.022 (3)	0.012 (2)	0.007 (2)	0.002 (2)
C15	0.030 (3)	0.046 (3)	0.023 (3)	0.012 (3)	0.007 (2)	0.003 (2)
C16	0.020 (2)	0.021 (2)	0.021 (2)	0.010 (2)	0.007 (2)	0.005 (2)
C17	0.051 (4)	0.046 (4)	0.052 (4)	0.017 (3)	0.031 (3)	0.021 (3)

### Geometric parameters (Å, °)

**Table:**

Cu1—N1	1.966 (4)	N7—H22	0.96 (2)
Cu1—O1	1.986 (3)	N7—H23	0.95 (2)
Cu1—N2	1.991 (4)	C1—C2	1.406 (8)
Cu1—O2	2.000 (3)	C1—H1	0.9500
Cu1—O3	2.252 (4)	C2—C3	1.382 (9)
Cu1—O4	2.610 (4)	C2—H2	0.9500
Cu2—N5	1.955 (4)	C3—C4	1.362 (8)
Cu2—O7	1.966 (3)	C3—H3	0.9500
Cu2—O8	1.981 (3)	C4—C5	1.397 (7)
Cu2—N6	1.994 (4)	C4—H4	0.9500
Cu2—O9	2.337 (3)	C5—C6	1.458 (8)
Cu2—O10	2.541 (4)	C6—C7	1.514 (7)
O1—C8	1.254 (5)	C7—H7A	0.9800
O2—C8^i^	1.258 (5)	C7—H7B	0.9800
O3—H18	0.96 (2)	C7—H7C	0.9800
O3—H19	0.95 (2)	C8—O2^i^	1.258 (5)
O4—N4	1.248 (5)	C8—C8^i^	1.527 (9)
O5—N4	1.227 (6)	C9—C10	1.386 (7)
O6—N4	1.260 (5)	C9—H9	0.9500
O7—C16	1.262 (5)	C10—C11	1.378 (8)
O8—C16^ii^	1.258 (5)	C10—H10	0.9500
O9—C17	1.409 (6)	C11—C12	1.384 (8)
O9—H24	0.8400	C11—H11	0.9500
O10—N8	1.237 (5)	C12—C13	1.398 (7)
O11—N8	1.228 (6)	C12—H12	0.9500
O12—N8	1.247 (6)	C13—C14	1.457 (7)
N1—C1	1.315 (7)	C14—C15	1.508 (7)
N1—C5	1.356 (6)	C15—H15A	0.9800
N2—C6	1.273 (7)	C15—H15B	0.9800
N2—N3	1.360 (6)	C15—H15C	0.9800
N3—H20	0.95 (2)	C16—O8^ii^	1.258 (5)
N3—H21	0.97 (2)	C16—C16^ii^	1.520 (9)
N5—C9	1.313 (7)	C17—H17A	0.9800
N5—C13	1.381 (6)	C17—H17B	0.9800
N6—C14	1.277 (6)	C17—H17C	0.9800
N6—N7	1.381 (6)		
			
N1—Cu1—O1	174.12 (15)	O11—N8—O10	120.4 (5)
N1—Cu1—N2	80.65 (18)	O11—N8—O12	120.7 (5)
O1—Cu1—N2	95.44 (16)	O10—N8—O12	118.9 (5)
N1—Cu1—O2	98.76 (16)	N1—C1—C2	121.7 (6)
O1—Cu1—O2	84.01 (13)	N1—C1—H1	119.2
N2—Cu1—O2	166.68 (14)	C2—C1—H1	119.2
N1—Cu1—O3	86.33 (15)	C3—C2—C1	117.6 (6)
O1—Cu1—O3	98.48 (13)	C3—C2—H2	121.2
N2—Cu1—O3	95.43 (16)	C1—C2—H2	121.2
O2—Cu1—O3	97.82 (14)	C4—C3—C2	120.4 (5)
N1—Cu1—O4	83.01 (14)	C4—C3—H3	119.8
O1—Cu1—O4	91.90 (12)	C2—C3—H3	119.8
N2—Cu1—O4	78.20 (14)	C3—C4—C5	119.7 (6)
O2—Cu1—O4	88.50 (12)	C3—C4—H4	120.1
O3—Cu1—O4	168.33 (13)	C5—C4—H4	120.1
N5—Cu2—O7	177.14 (15)	N1—C5—C4	119.5 (5)
N5—Cu2—O8	95.30 (15)	N1—C5—C6	115.2 (4)
O7—Cu2—O8	85.09 (13)	C4—C5—C6	125.3 (5)
N5—Cu2—N6	81.51 (17)	N2—C6—C5	114.2 (4)
O7—Cu2—N6	97.92 (15)	N2—C6—C7	123.9 (5)
O8—Cu2—N6	175.14 (15)	C5—C6—C7	121.8 (5)
N5—Cu2—O9	89.35 (14)	C6—C7—H7A	109.5
O7—Cu2—O9	93.43 (13)	C6—C7—H7B	109.5
O8—Cu2—O9	95.95 (13)	H7A—C7—H7B	109.5
N6—Cu2—O9	87.70 (14)	C6—C7—H7C	109.5
N5—Cu2—O10	85.40 (14)	H7A—C7—H7C	109.5
O7—Cu2—O10	91.78 (13)	H7B—C7—H7C	109.5
O8—Cu2—O10	87.50 (13)	O1—C8—O2^i^	126.0 (4)
N6—Cu2—O10	88.58 (14)	O1—C8—C8^i^	117.4 (5)
O9—Cu2—O10	173.97 (12)	O2^i^—C8—C8^i^	116.6 (5)
C8—O1—Cu1	111.0 (3)	N5—C9—C10	122.3 (5)
C8^i^—O2—Cu1	110.9 (3)	N5—C9—H9	118.9
Cu1—O3—H18	106 (4)	C10—C9—H9	118.9
Cu1—O3—H19	126 (3)	C11—C10—C9	118.0 (5)
H18—O3—H19	92 (5)	C11—C10—H10	121.0
N4—O4—Cu1	110.5 (3)	C9—C10—H10	121.0
C16—O7—Cu2	110.2 (3)	C10—C11—C12	120.7 (5)
C16^ii^—O8—Cu2	110.3 (3)	C10—C11—H11	119.7
C17—O9—Cu2	119.5 (3)	C12—C11—H11	119.7
C17—O9—H24	109.5	C11—C12—C13	119.1 (5)
Cu2—O9—H24	116.3	C11—C12—H12	120.4
N8—O10—Cu2	113.3 (3)	C13—C12—H12	120.4
C1—N1—C5	121.1 (5)	N5—C13—C12	118.7 (5)
C1—N1—Cu1	125.4 (4)	N5—C13—C14	115.4 (4)
C5—N1—Cu1	113.4 (3)	C12—C13—C14	125.9 (5)
C6—N2—N3	121.3 (5)	N6—C14—C13	114.4 (4)
C6—N2—Cu1	116.4 (4)	N6—C14—C15	123.1 (5)
N3—N2—Cu1	122.0 (3)	C13—C14—C15	122.5 (4)
N2—N3—H20	110 (5)	C14—C15—H15A	109.5
N2—N3—H21	108 (5)	C14—C15—H15B	109.5
H20—N3—H21	116 (7)	H15A—C15—H15B	109.5
O5—N4—O4	120.6 (5)	C14—C15—H15C	109.5
O5—N4—O6	120.2 (5)	H15A—C15—H15C	109.5
O4—N4—O6	119.2 (4)	H15B—C15—H15C	109.5
C9—N5—C13	121.2 (4)	O8^ii^—C16—O7	125.8 (4)
C9—N5—Cu2	126.3 (3)	O8^ii^—C16—C16^ii^	116.7 (5)
C13—N5—Cu2	112.6 (3)	O7—C16—C16^ii^	117.6 (5)
C14—N6—N7	121.9 (4)	O9—C17—H17A	109.5
C14—N6—Cu2	115.9 (4)	O9—C17—H17B	109.5
N7—N6—Cu2	121.5 (3)	H17A—C17—H17B	109.5
N6—N7—H22	106 (4)	O9—C17—H17C	109.5
N6—N7—H23	111 (4)	H17A—C17—H17C	109.5
H22—N7—H23	119 (5)	H17B—C17—H17C	109.5

Symmetry codes: (i) −*x*, −*y*−1, −*z*−1; (ii) −*x*+2, −*y*, −*z*.

### Hydrogen-bond geometry (Å, °)

**Table:**

*D*—H···*A*	*D*—H	H···*A*	*D*···*A*	*D*—H···*A*
O3—H18···O11^iii^	0.96 (2)	2.11 (3)	3.048 (7)	164 (5)
O3—H18···O12^iii^	0.96 (2)	2.30 (4)	3.084 (6)	138 (5)
O3—H18···N8^iii^	0.96 (2)	2.53 (2)	3.471 (6)	167 (5)
O3—H19···O9^iv^	0.95 (2)	1.85 (2)	2.777 (5)	165 (5)
N3—H20···O1	0.95 (2)	2.53 (7)	3.014 (6)	112 (5)
N3—H21···O12	0.97 (2)	2.44 (7)	3.150 (7)	130 (7)
N7—H22···O6	0.96 (2)	2.35 (4)	3.131 (6)	138 (5)
N7—H23···N3	0.95 (2)	2.43 (5)	3.196 (7)	138 (5)
N7—H23···O7	0.95 (2)	2.59 (6)	3.091 (5)	113 (5)
O9—H24···O6^v^	0.84	1.89	2.731 (5)	174
O9—H24···N4^v^	0.84	2.57	3.367 (6)	158
O9—H24···O5^v^	0.84	2.56	3.171 (6)	130

Symmetry codes: (iii) −*x*+1, −*y*, −*z*; (iv) *x*−1, *y*, *z*; (v) −*x*+1, −*y*−1, −*z*−1.
